# Cost utility analysis of reduced intensity hematopoietic stem cell transplantation in adolescence and young adult with severe thalassemia compared to hypertransfusion and iron chelation program

**DOI:** 10.1186/1472-6963-13-45

**Published:** 2013-02-05

**Authors:** Rosarin Sruamsiri, Nathorn Chaiyakunapruk, Samart Pakakasama, Somtawin Sirireung, Nintita Sripaiboonkij, Udomsak Bunworasate, Suradej Hongeng

**Affiliations:** 1 Center of Pharmaceutical Outcomes Research, Department of Pharmacy Practice, Faculty of Pharmaceutical Sciences, Naresuan University, Phitsanulok, Thailand; 2 School of Population Health, University of Queensland, Brisbane, Australia; 3 School of Pharmacy, University of Wisconsin-Madison, Madison, WI, USA; 4 Jeffrey Cheah School of Medicine and Health Sciences, Monash University Sunway campus, Monash, Malaysia; 5 Department of Pediatrics, Faculty of Medicine, Ramathibodi Hospital, Mahidol University, Bangkok, Thailand; 6 Cancer registry unit, health case management services, Ramathibodi Hospital, Mahidol University, Bangkok, Thailand; 7 Department of Medicine, Faculty of Medicine, Chulalongkorn Memorial Hospital, Chulalongkorn University, Bangkok, Thailand

**Keywords:** Cost-utility analysis, Reduced intensity transplantation, Thalassemia, Adolescence, Adult

## Abstract

**Background:**

Hematopoieticic stem cell transplantation is the only therapeutic option that can cure thalassemia disease. Reduced intensity hematopoietic stem cell transplantation (RI-HSCT) has demonstrated a high cure rate with minimal complications compared to other options. Because RI-HSCT is very costly, economic justification for its value is needed. This study aimed to estimate the cost-utility of RI-HSCT compared with blood transfusions combined with iron chelating therapy (BT-ICT) for adolescent and young adult with severe thalassemia in Thailand.

**Methods:**

A Markov model was used to estimate the relevant costs and health outcomes over the patients’ lifetimes using a societal perspective. All future costs and outcomes were discounted at a rate of 3% per annum. The efficacy of RI-HSCT was based a clinical trial including a total of 18 thalassemia patients. Utility values were derived directly from all patients using EQ-5D and SF-6D. Primary outcomes of interest were lifetime costs, quality adjusted life-years (QALYs) gained, and the incremental cost-effectiveness ratio (ICER) in US ($) per QALY gained. One-way and probabilistic sensitivity analyses (PSA) were conducted to investigate the effect of parameter uncertainty.

**Results:**

In base case analysis, the RI-HSCT group had a better clinical outcomes and higher lifetime costs. The incremental cost per QALY gained was US $ 3,236 per QALY. The acceptability curve showed that the probability of RI-HSCT being cost-effective was 71% at the willingness to pay of 1 time of Thai Gross domestic product per capita (GDP per capita), approximately US $ 4,210 per QALY gained. The most sensitive parameter was utility of severe thalassemia patients without cardiac complication patients.

**Conclusion:**

At a societal willingness to pay of 1 GDP per capita, RI-HSCT was a cost-effective treatment for adolescent and young adult with severe thalassemia in Thailand compared to BT-ICT.

## Background

Thalassemia is one of the important hematological diseases in Thailand. Thalassaemia patients have produced abnormal haemoglobin, leading to anaemia.The prevalence of thalassemia carriers in Thailand was reported as 30%-40%. About 1% of Thai population, approximately 65,000 people, have thalassemia [1]. The standard treatment of severe thalassemia is regular blood transfusion combined with iron chelating therapy (BT-ICT) to prevent iron overload. BT-ICT treatment has to be given subcutaneously for 8 to12 hours per days, 5 to 7 days per week. This arduous treatment regimen and the high cost can lead to poor compliance and difficulty in patients’ life which could affect the effectiveness of the treatment. Adult thalassemia patients (age 17 or older) have more advanced disease and treatment related organ complications mainly due to prolonged exposure to iron overload [2].

At present, the only curative treatment existing for severe thalassemic patients was hematopoietic stem cell transplantation (HSCT) [2]. However; HSCT was used with limitations because of a number of reasons. Compared to younger patients, the transplantation associated mortality and rejection rates, in patients aged more than 17 years old with iron-overload related organ damage, are as high as 40% and 16%, respectively [2]. Moreover, the percentages of human leukocyte antigen (HLA)-matched donor were as low as 25% for patient’s relative donor and 1 out of 50,000 in the unrelated donors [3-5].

Reduced intensity hematopoietic stem cell transplantation (RI-HSCT) is a new preparative regimen for high risk thalassemia patients especially adolescent and adult. RI-HSCT has been demonstrated to be an effective strategy to reduce the toxicity of transplantation with low incidence of graft rejection. This novel approach is a promising alternative for reducing the risk of life-threatening complications and increasing the number of patients successfully cured with an allograft [6]. Reduced intensity preparative regimens are demonstrated with lower morbidity and mortality after transplantation [7]. In Thailand, Hongeng et al. [8] performed RI-HSCT in 8 patients with Class 3 Lucarelli. Seven patients received peripheral blood stem cells (PBSCs) from HLA-matched sibling donors while one patient received from haploidentical mother. An engraftment of donor cells was observed in all patients. Six out of 8 patients had stable full donor engraftment. There were no deaths or grade 3–4 acute graft-versus-host disease (GvHD) among these patients.

Although RI-HSCT has demonstrated efficacy, its procedure is complex and incurs high expenditure especially during the first three years of treatment. Given the high cost of RI-HSCT, there remains an important question to be answered from a policy maker perspective whether RI-HSCT is worth the money spent and increase quality of life of patients. This study examines the potential cost-utility of RI-HSCT compared to BT-ICT in the treatment of patients with severe adolescent and young adult thalassemia.

## Methods

### Overall description of cost-utility analysis methodology

We used a markov model to simulate severe thalassemia patients receiving RI-HSCT compared to BT-ICT, which is a standard practice of thalassemia treatment. The study was undertaken using a societal perspective as recommended by the Thailand’s health technology assessment guidelines [9]. We performed a cost-utility analysis with incremental cost per QALY gained.

The model simulates for the life time horizon. Clinical effectiveness and economic outcomes was directly obtained from an ongoing clinical investigation of RI-HSCT in 18 thalassemia patients in Thailand. The simulated cohort’s characteristics of the model were based on the characteristics of 18 patients. Ethical approval was granted by the Committee on Human Rights Related to Research Involving Human Subjects, Mahidol University.

### Economic model

**Figure 1:**
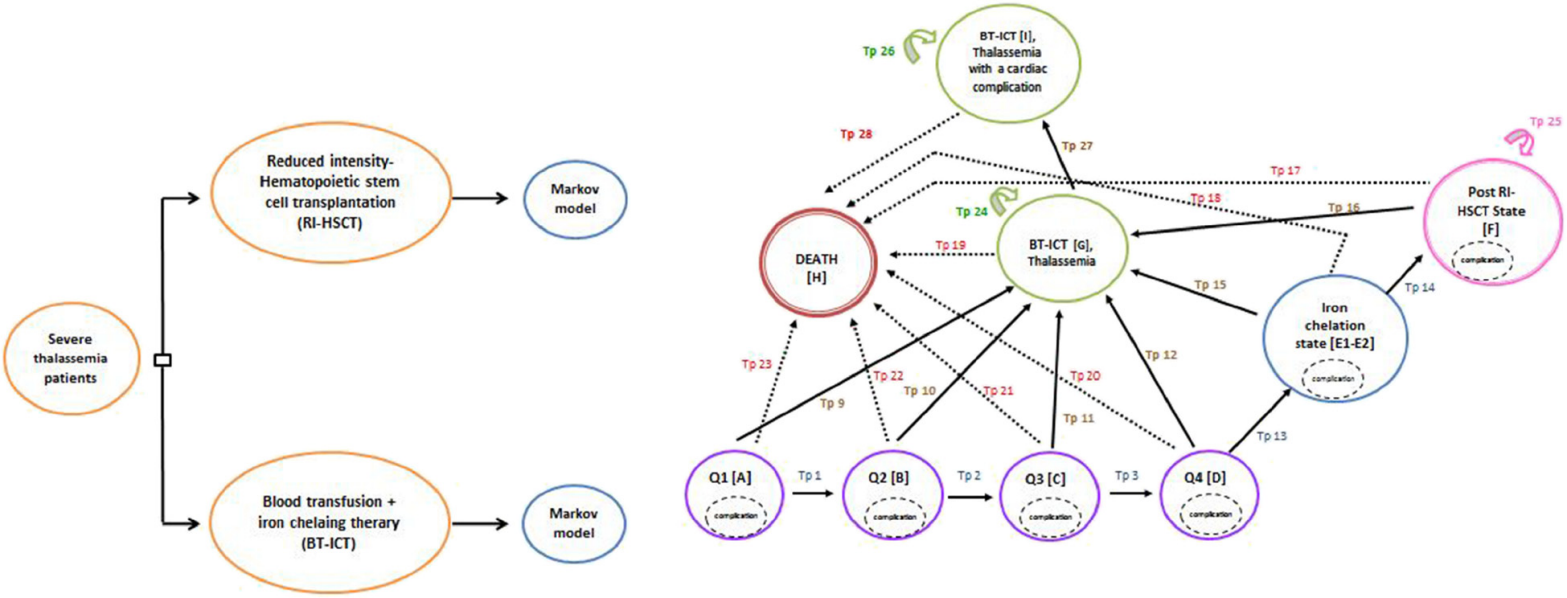
**Schematic diagram of the markov model.** Each thalassemia patient has two treatment options (RI-HSCT and BT-ICT) The Markov model consists of eight health states and patients receiving RI-HSCT can transition through each of these health states whereas BT-ICT patients can be in either alive BT-ICT state or death state. The cycle length is one year with a 99-year time horizon. RI-HSCT: Reduced intensity hematopoietic stem cell transplantation; BT-ICT: Blood transfusion combined with iron chelating therapy.

A Markov model, as shown in Figure 1, was used to estimate the relevant costs and health outcomes. The health states of patients assigned to RI-HSCT consisted of eight states, which were categorized into five groups to mimic the natural history of thalassemia and clinical practice; (i) the first year of RI-HSCT consisted of four states [Q_1_-Q_4_], with the length of three months each. The first six months [Q_1_-Q_2_] is the period with the highest costs, highest graft rejections and worst quality of life (QoL). We assumed that the rejection rate in this period is consistent. The remaining six months [Q_3_-Q_4_] is when the patients needed close follow up in order to monitor side effects, resulting in more frequent visits. No graft rejection occured in this period; (ii) The second and third year of RI-HSCT [Iron chelation] was when patients had higher costs due to either regular phlebotomy or chelation therapy and immunosuppressive therapy; (iii) following years after successful RI-HSCT [Post RI-HSCT] (iv) RI-HSCT failure resulting in a switch to BT-ICT. After graft rejection, patients were to receive blood transfusions and subcutaneous infusion iron chealtion therapy only. Since cardiac disease is the major cause of death, we constructed the BT-ICT health state with two sub-health states: thalassemia patients without complication and thalassemia patients with cardiac complication; and (v) death Blood transfusion dependent patients had two health states (i.e. BT-ICT and death). The arrows represent the possible transitions from one state to the other. Treatment complications were included within all health states as they typically took far less than one year to resolve. The simulation estimated the costs and health outcomes over a lifetime time horizon. All transition probabilities, costs and outcomes variables are shown in Table 1.

**Table 1:** Input parameters used in the model

** Variable**	**Mean (SE)**	**Sources**
**Clinical Variable (Transitional Probability)**		
***RI-HSCT***		
Q1 state --> Death	Vary	Cohort, MOPH [10]
Q1 state --> BT-ICT	0.00003	Cohort
Q2 state --> Death	Vary	Cohort, MOPH [10]
Q2 state --> BT-ICT	0.0003	Cohort
Q3 state --> Death	Age-specific Mortality	MOPH [10]
Q3 state --> BT-ICT	0.00	Assumption
Q4 state --> Death	Age-specific Mortality	MOPH [10]
Q4 state --> BT-ICT	0.00	Assumption
Iron chelation state --> Death	Age-specific Mortality	MOPH [10]
Iron chelation state--> BT-ICT	0.00	Assumption
Post RI-HSCT --> Death	Age-specific Mortality	MOPH [10]
Post RI-HSCT--> BT-ICT	0.00	Assumption
***BT-ICT (Severe Thalassemia patients)***		
BT-ICT Thal --> BT-ICT Thal with cardiac	0.0114	Borgna-Pignatti C et al.[11]
BT-ICT Thal --> Death	Vary	MOPH [10], Delea et al. [12], Gabutti & Piga [13]
BT-ICT Thal with cardiac --> Death	Vary	MOPH [10] and Chouliaras G [14]
Cost Variable (convert to US $) *,!		
***RI-HSCT***		
*Pre-BMT*		
Direct Medical Cost	1,330 (438)	Hospital Database^#^
Direct Non Medical Cost	75 (21)	Survey
Indirect Cost	175 (42)	Survey
*Q1 state*		
Direct Medical Cost	1,226 (169)	Hospital Database^#^
Direct Non Medical Cost	458 (51)	Survey
Indirect Cost	945 (105)	Survey
*Q2 state*		
Direct Medical Cost	1,710 (381)	Hospital Database^#^
Direct Non Medical Cost	335 (41)	Survey
Indirect Cost	691 (85)	Survey
*Q3 state*		
Direct Medical Cost	1,628 (538)	Hospital Database^#^
Direct Non Medical Cost	259 (30)	Survey
Indirect Cost	535 (62)	Survey
*Q4 state*		
Direct Medical Cost	878 (282)	Hospital Database^#^
Direct Non Medical Cost	253 (32)	Survey
Indirect Cost	522 (66)	Survey
*Iron chelation state*		
Direct Medical Cost	1,120 (238)	Hospital Database^#^
Direct Non Medical Cost	539 (112)	Survey
Indirect Cost	1,111 (230)	Survey
*Post RI-HSCT state*		
Direct Non Medical Cost	1,548 (411)	Hospital Database^#^
Direct non Medical Cost	299 (146	Survey
Indirect Cost	617 (301)	Survey
***BT-ICT (Severe Thalassemia patients)***		
Severe thalassemia patients without cardiac complications		
Direct Medical Cost	1,187 (137)	Riewpariboon et al. [28]
Direct Non Medical Cost	1,240 (232)	Leelahavaring et al. [29]
Indirect Cost	636 (221)	Leelahavaring et al. [29]
Severe thalassemia patients with cardiac complications		
Direct Medical Cost	1,187 (137)	Riewpariboon et al. [28]
Direct Non Medical Cost	1,240 (232)	Leelahavaring et al. [29]
Indirect Cost	636 (221)	Leelahavaring et al. [29]
**Effectiveness Variable**		
***RI-HSCT***		
Q1 state	0.59 (0.30)	Survey
Q2 state	0.59 (0.30)	Survey
Q3 state	0.59 (0.30)	Survey
Q4 state	0.59 (0.30)	Survey
Iron chelation state	0.88 (0.06)	Survey
Post-RI-HSCT	0.90 (0.05)	Survey
***BT-ICT (Severe Thalassemia patients)***		
Severe thalassemia patients without cardiac complications	0.61 (0.03)	Osborne et al. [33]
Severe thalassemia patients with cardiac complications	0.46 (0.02)	Osborne et al. [33]

RI-HSCT: Reduced intensity hematopoietic stem cell, BT-ICT: Blood transfusion combined with subcutaneous iron chelating therapy, Pre-BMT: Pre-bone marrow transplantation.

* ($US, year 2011 value).

^#^ Charges were converted to cost using a cost-to-charge ratio of 1.37 which was derived from Ramathibodi hospital for base-case analysis and 0.8-1.5 [27] for sensitivity analysis.

^!^ Costs in Q_1_-Q_4_ is calculated for 3 month period, while costs beyond Q4 are on an annual basis.

### Description of reduced intensity hematopoietic stem cell (RI-HSCT)

The first cohort of 8 patients received conditioning regimen as described previously [8] and the remaining 10 patients received a new reduced intensity conditioning regimen consisting of intravevous busulfan 130 mg/m^2^ for 4 days, fludarabine 35 mg/m^2^ for 6 days and antithymocyte globulin (Thymoglobulin®) 1.5 mg/kg for 4 days and they also received fludarabine 30 mg/m^2^ for 5 days and dexamethasone 25 mg/m^2^ for 5 days 1 month prior to transplant for immunosuppression rationale. The reduced intensity regimen was designed for patients who were classified as having Lucarelli class 3. All these patients were given hydroxyurea 20 mg/kg/d ≥ 3 months before BMT to decrease erythroid expansion and thus prevent graft rejection [19-21].

Stem cell grafts are prepared by the following procedures. T-cell nondepleted peripheral blood stem cell graft was infused on day 0. Only 1 patient received purified CD34^+^ cells that were derived from 2 antigens and HLA-mismatched maternal peripheral blood stem cells with additional CD3^+^ cells to a total of 1 × 10^5^ CD3^+^ cells/kg [21]. These purified CD34^+^ cells were obtained with Clinimacs (Miltenyi, Bergisch, Germany). All donors received subcutaneous granulocyte colony-stimulating factor 10 μg/kg/d for 4 days before leukapheresis procedures.

For graft-versus-host disease (GVHD) prophylaxis, Three days before transplantation, patients received cyclosporin at a dose that was adjusted to achieve a plasma concentration of 250–350 ng/mL or tacrolimus at a dose that was adjusted to achieve a plasma concentration of 5–15 ng/mL. All received a short course of methotrexate [22]. Engraftment Criteria; 3 consecutive days with an absolute neutrophil count >.5 × 10^9^/L or 7 consecutive days with a platelet count >20 × 10^9^/L without transfusion were taken as evidence of engraftment.

In order to prevent *Pneumocystis carinii* pneumonia*,* patients received a preventive regimen of trimethoprim-sulfamethoxazole (cotrimoxazole) at 5 mg/kg/d divided into 2 doses for 3 days a week. Cytomegalovirus (CMV) and Ebstein-Barr virus (EBV) reactivations were monitored by CMV and EBV viral loads. Patients with a CMV viral load >10^3^ copies/mL were treated with ganciclovir [23] and intravenous immunoglobulin [23] and patients with an EBV viral load >10^3^ copies/mL were treated with rituximab [24].

### Effectiveness data

#### Effectiveness of RI-HSCT

A total of 18 patients receiving RI-HSCT at Ramathibodi hospital between 2003 and 2011 were used a source of clinical effectiveness of the intervention. Five cases were patients with homozygous β-thalassemia while the remaining 13 were β-thalassemia/hemoglobin E. The time to death and time to failure were directly derived from this cohort using a parametric survival-time model with Exponential Regression [25]. This is to yield which is the cumulative hazard.

$$H_{t}=\lambda t^{\gamma }$$

where *H*_*t*_ is the cumulative hazard; *λ* (lambda) is the scale parameter; *t* is time in days; and ancillary or *γ* (gamma) is the shape parameter that describes the instantaneous hazard rate (*h*_*t*_).

The survival function, (*S*_*t*_) which describes the probability of survival at time *t*

$$S_{t}=\exp\left(-h_{t}\right)$$

The transitional probability of the event of interest (death or failure) Probability of RI-HSCT is calculated using *S*_*t*_ and *S*_*t* − *u*_ being estimated from the following formula (where *u* is the cycle length of the model)

$$P=1-\frac{S_{t}}{S_{t-u}}$$

Due to the limited number of patients, the probability of death derived from RI-HSCT patients would not reflect the true mortality rate. We calculated the probability to death in terms of Thai population by converting the probability of death from the cohort into rate of death, then adding the age-specific Thai mortality rate [10] and convert the result back into probability in order to reflect all population. Because there were no cases of failure of RI-HSCT after six months of treatment, we assumed zero probability of failure in subsequent periods. Based on our cohort, 2 of 18 patients were failed of RI-HSCT. One was failed within 50 days, while another was 131 days. Thus, we applied the zero probability of failure in the model after 180 days of RI-HSCT.

#### Effectiveness of BI-ICT

For patients receiving BT-ICT, we estimated the risk of death based on the multiplication of Thai age-specific mortality [10] and relative risk of 3.9. This relative risk value was derived by Delea et al. [12] using the cohort data of 257 thalassaemia patients reported in a study by Gabutti and Piga et al. [13]. The probability of transitioning from thalassaemia without cardiac complication to thalassaemia with a cardiac complication was derived from a cohort study of 1073 thalassaemia patients [11]. We converted annual risk of having cardiac complications from a heart failure-free probability. For the risk of death for patients with cardiac complications, we combined the age-specific mortality rate for patients with thalassaemia without complications and the age specific mortality rate related to cardiac diseases. The latter mortality rate was obtained from a cohort study that followed up 648 thalassaemia in order to determine the risk of cardiac related death almost 28 years [14]. Based on the cohort of 18 patients, no one received RI-HSCT more than once. Therefore, in the model it was assumed that if the RI-HSCT failed, patients would receive BT-ICT for the rest of their lives.

### Cost data

As this study was undertaken using the societal perspective, each group consisted of direct medical costs, direct non medical costs, and indirect costs. All costs were converted and reported in 2011 US $ (US $1= 30.50 THB [26]) using the consumer price index (CPI) [15]. We divided costs for RI-HSCT groups into 2 parts which were cost of pre-bone marrow transplantation (Pre-BMT) and costs of transplantation and follow up. Direct medical costs were estimated using micro-costing approach [16]. Direct medical costs for pre-bone marrow transplantation included laboratory tests and investigation from patients and donors were obtained from hospital databases. Direct medical costs for transplantation and follow up were calculated starting from the first date of patient’s admission during pre-transplantation management. Costs were divided in line with the heath states in the model, for example; costs in the first year [Q1-Q4] were estimated in 3 month-period, while costs of iron chelation and post RI-HSCT were calculated annually. Direct medical costs for RI-HSCT group were obtained from two data sources. First, costs of patients receiving RI-HSCT were retrieved from a hospital database at a teaching hospital between 2003 and 2011. Cost to charge ratio was used to convert hospital charges to cost by multiplying charges to cost to charge ratio. Conversion of hospital charge to costs is a reasonable trade-off between accuracy and the resources used for costing [16]. Charges were converted to cost using a cost-to-charge ratio of 1.37 which was derived from Ramathibodi hospital for base-case analysis and 0.8-1.5 [27] for sensitivity analysis. Other direct medical costs for example: out of pocket expenses which related to medications were collected using questionnaires. Both direct non-medical and indirect costs for RI-HSCT groups were collected by interviewing severe thalassemia patients and their caregivers at Ramathibodi hospital after obtaining informed consent. Eighteen RI-HSCT patients were interviewed by questionnaire. Direct non-medical costs included; costs of transportation, meals, accommodation, facilities, other self care expenses and informal care. Indirect costs referred to productivity losses due to sick leave. Resource cost parameters are presented in Table 1.

Because of the lack of separated costs data for severe thalassemia patients with or without cardiac complications, we decided to use costs of severe thalassemia patients based on a study of Leelahavarong et al. and Riewpariboon et al. as cost inputs in our model. The costs for severe thalassemia patients were obtained from two literature studies. First, direct medical costs were derived from a cost analysis by Riewpaiboon et al. [28] which is the largest cost-of-illness of thalassemia patients study in Thailand. The study population consisted of 124 severe thalassemia patients. Mean direct medical costs per year were US $ 1,187 (SE = 137). For direct non-medical and indirect costs, we obtained data from Leelahavarong et al. [29] which were collected from severe thalassemia patients (same as our study). This study interviewed 28 severe thalassemia patients who received hyper-blood transfusion and iron chelation treatment at Ramathibodi hospital using questionnaire. Direct non-medical costs were estimated US $ 1,240 (SE = 232) and US $ 636 (SE = 221) for indirect costs.

### Utility data

We measured health-related quality of life of RI-HSCT patients using SF-36V2 [Thai version of the SF-36 (short-form survey) version 2.0] and EQ5D (EuroQoL EQ-5D). In Thailand, the validated official Thai version of EQ-5D is available. The answer from Thai version of EQ-5D questionnaire can be converted into 0–1 utility score by Thai preference score for EQ-5D health state [30]. Because of its practicality and accepted reliability, EQ-5D is the recommended utility measurement method in guideline of Thailand’s health technology assessment [9]. The other advantage of EQ-5D is that it is easier for patients to answer the questionnaires compared to decision making in preference elicitation techniques. We used utility derived from EQ-5D as main analysis and SF-36 as sensitivity analysis. The SF-36 is a multipurpose, short-form health survey with only 36 questions. It yields an eight-scale profile of scores as well as physical and mental health summary measures [17]. The questionnaire consists of 36 items that cover 8 aspects of health-related quality of life including physical functioning, role limitations due to physical problems, social functioning, bodily pain, general mental health, role limitation due to emotional problems, vitality (energy/fatigue) and general health perceptions. SF-36V2 [31] (Thai version) is a widely used instrument with high validity and reliability. The utility scores were estimated via SF-6D derived from the SF-36. SF-6D was converted using SF-36 data at the individual level to single utility score using regression model [32]. We adopted the approach of Delea et al. [12] in deriving utility values for severe thalassemia patients without cardiac complication from the literature. The utility value was 0.61 (0.66). These values were taken from an Australian study [33], using a time trade-off (TTO) exercise to elicit utility values from a sample of 120 individuals of various demographic characteristics. For health state with cardiac complications, we also adopted the approach of Delea et al. [12] and deducted a utility of 0.15. This value was obtained from a cohort study which estimatd a disutility value of heart failure based on 1365 random samples of US adults in a community [34].

### Cost-utility analysis

Primary outcomes of interest were lifetime costs, QALYs gained, and ICER in US ($) per QALY gained. For base-case analysis, we calculated the expected lifetime costs and outcomes from each group of treatment. All future costs and outcomes were discounted at a rate of 3% per annum [35]. The results were presented as ICER for RI-HSCT *vs* BT-ICT

$$\mathrm{ICER}=\frac{\mathrm{Cos}t_{\mathrm{RI}-\mathrm{HSCT}}-\mathrm{Cos}t_{\mathrm{BT}-\mathrm{ICT}}}{\mathrm{QALY}s_{\mathrm{RI}-\mathrm{HSCT}}-\mathrm{QALY}s_{\mathrm{BT}-\mathrm{ICT}}}$$

The interpretation of cost-effectiveness of our findings is based on the statement of the Subcommittee for Development of the National List of Essential Drugs and the Subcommittee for Development of the Health Benefit Package and Service Delivery of the NHSO in 2007. The subcommittee set up the societal willingness to pay (WTP) threshold in Thailand to one to three times per capita Gross Domestic Product (GDP) [36], approximating US $ 4,210 (128,403 THB) to US $ 12,263 (385,209 THB) per QALY gained in 2010 [18]. These values are in accordance with the recommendations of the Commission on Macroeconomics and Health of World Health Organization, suggesting that health technologies with the incremental cost-effectiveness ratio (ICER) below the per capita GDP are considered very cost-effective, while those between one and three times per capita GDP being cost-effective, and ICER above three times per capita GDP indicate that a health technology is not cost-effective [37].

### Sensitivity analysis

One-way sensitivity analyses were performed to investigate the effects of altering parameters within plausible ranges including survival parameters, time to failure parameters, all costs, utilities, costs to charge ratio and discounting rate. The results of one-way sensitivity were presented using a tornado diagram. For the sensitivity analysis of discounting rate, we used the recommendation from WHO [35] including 0% discount costs and health outcomes, 0% discount health outcomes and 6% discount costs, and 6% discount cost and health outcomes. In addition, a probabilistic sensitivity analysis (PSA) was conducted to examine the effect of all parameter uncertainty simultaneously using a Monte Carlo simulation performed by Microsoft Excel 2003 (Microsoft Corp., Redmond, WA) [25]. The distributions of each probability were assigned following [38]: (a.) probability and utility parameters, in which their values ranged between zero and one, were specified to beta-distributions, (b.) costs, their characters were positively skewed and values were above zero, were assigned to gamma-distributions, and (c.) survival parameters were given to a log-normal distribution. A Monte Carlo simulation was run for 1,000 sets of the simulation to give a range of values for total costs, outcomes, and ICERs. Results of the PSA were presented as cost-effectiveness acceptability curves. The expected net monetary benefit (NMB) was calculated for each WTP threshold in Thailand (one and three GDP per capita).

## Results and discussion

### Clinical effectiveness

**Figure 2:**
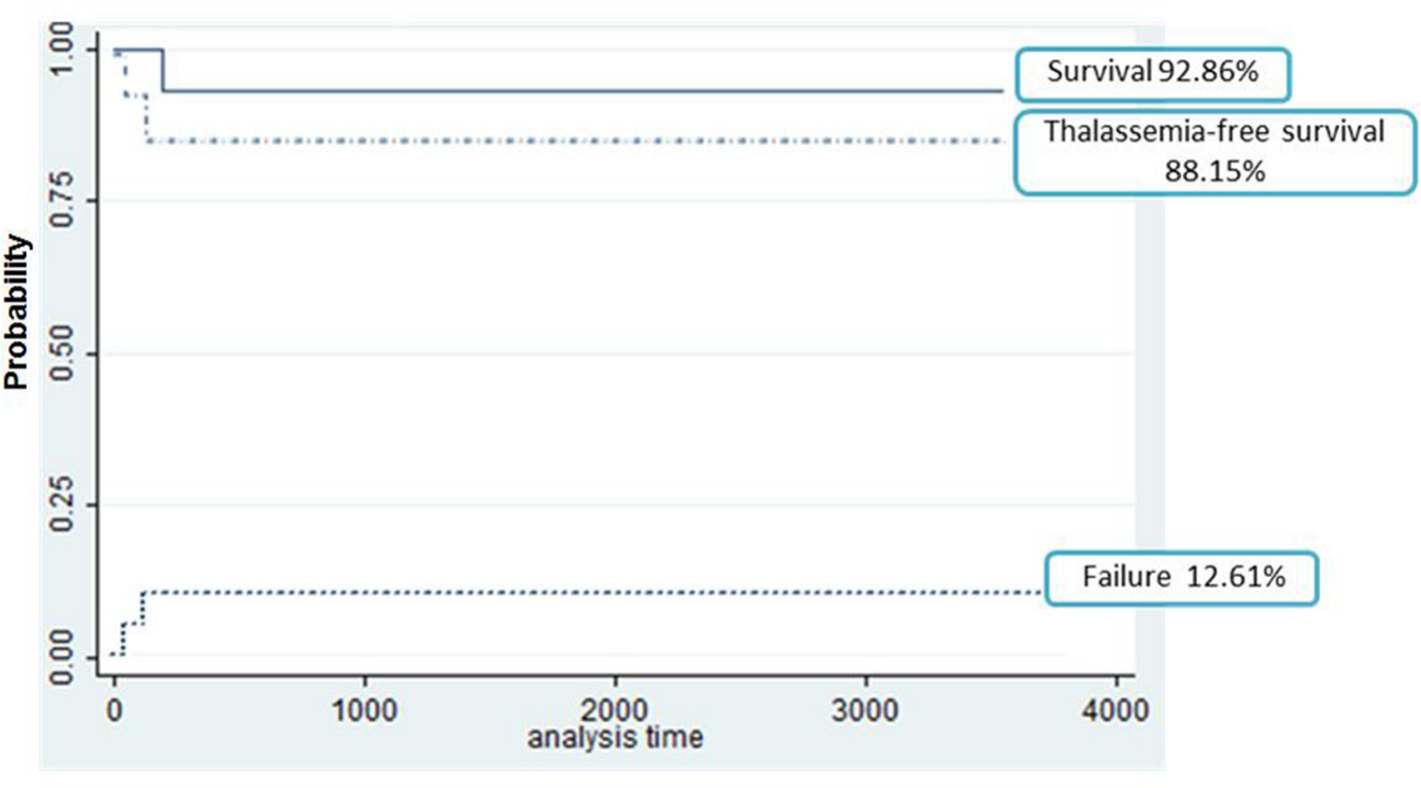
**Estimates of survival and graft rejection for 18 patients receiving RI-HSCT.** RI-HSCT: Reduced intensity hematopoietic stem cell transplantation.

A total of 18 patients receiving RI-HSCT were included in this study. Mean age was 13.7 years; range 9–18 years. Peripheral Blood Stem Cell (PBSC) was used as sources for stem cell transplantation for all patients. Patients were classified as class III according to Lucarelli or Pesaro classification [2]. Of 18 patients, two patients have comorbidity diseases (hypothyroid and diabetes). The average duration of follow-up is 2 years with the range from 1 to 8 years. Demographic description of these patients was shown in Table 2. Based on our cohort, 2 of 18 patients (11%) were failed of RI-HSCT. One was failed within 50 days, while another was 131 days. Only one patients died of transplantation-related complications (namely pulmonary infection).The Kaplan-Meier curve was displayed in Figure 2.

**Table 2:** Characteristics of 18 patients receiving RI-HSCT

** Variable**	**N (%)**
**Demographic characteristic**	
Age, years	
Mean + SD	13.7 + 2.7
Range	9 – 18
Female sex	11 (61.1)
Health Insurance	
CSMBS	7 (38.9)
UC	6 (33.3)
Out-of-pocket	4 (22.2)
Others	1 (5.6)
Education	
Lower than primary school	3 (16.7)
Primary school	1 (5.6)
Secondary school	7 (38.9)
College	2 (11.1)
Undergraduate school	5 (27.7)
**Transplant characteristic**	
Thalassemia type	
homozygous beta-thalassemia	5 (27.8)
homozygous beta-thalassemia/ Hemoglobin E	13 (72.2)
Stem cell source	
PBSC	18 (100)
Number of Donor	
Mean + SD	1.3 + 0.6
Range	1 – 3

CSMBS: Civil servant medical benefit scheme; UC: Universal coverage; PBSC: Peripheral blood stem cell.

### Cost-utility analysis

Our base-case analysis demonstrated the estimated life time cost for BT-ICT and RI-HSCT as US $ 73,928 (2,254,806 Thai baht) and US$ 114,000 (3,476,988 Thai baht), respectively, while the estimated QALYs were 14.11, and 26.49 QALYs, respectively. (Table 3) Compared to BT-ICT, the conventional therapy of severe thalassemia with hyper-blood transfusion and iron-chelating therapy, an incremental cost per QALY gained for RI-HSCT was US$ 3,236 per QALY.

**Table 3:** Results from base-case analysis

** Outcome measure**	**RI-HSCT**	**BT-ICT**	**RI-HSCT vs BT-ICT**
Costs*			
Direct Medical Cost	83,733.58	28,651.11	55,082.47
Direct Non Medical Cost	9,877.00	29,928.83	−20,541.83
Indirect Cost	20,389.58	15,347.89	5,041.69
**Total**	114,000.16	73,927.83	40,072.33
**QALYs**	26.50	14.11	12.38
**Cost per QALY gained**			3,236.37

QALY = Quality adjusted life year; RI-HSCT: Reduced intensity hematopoietic stem cell; BT-ICT: Blood transfusion combined with subcutaneous iron chelating therapy.

* ($US, year 2011 value).

### Sensitivity analysis

**Figure 3:**
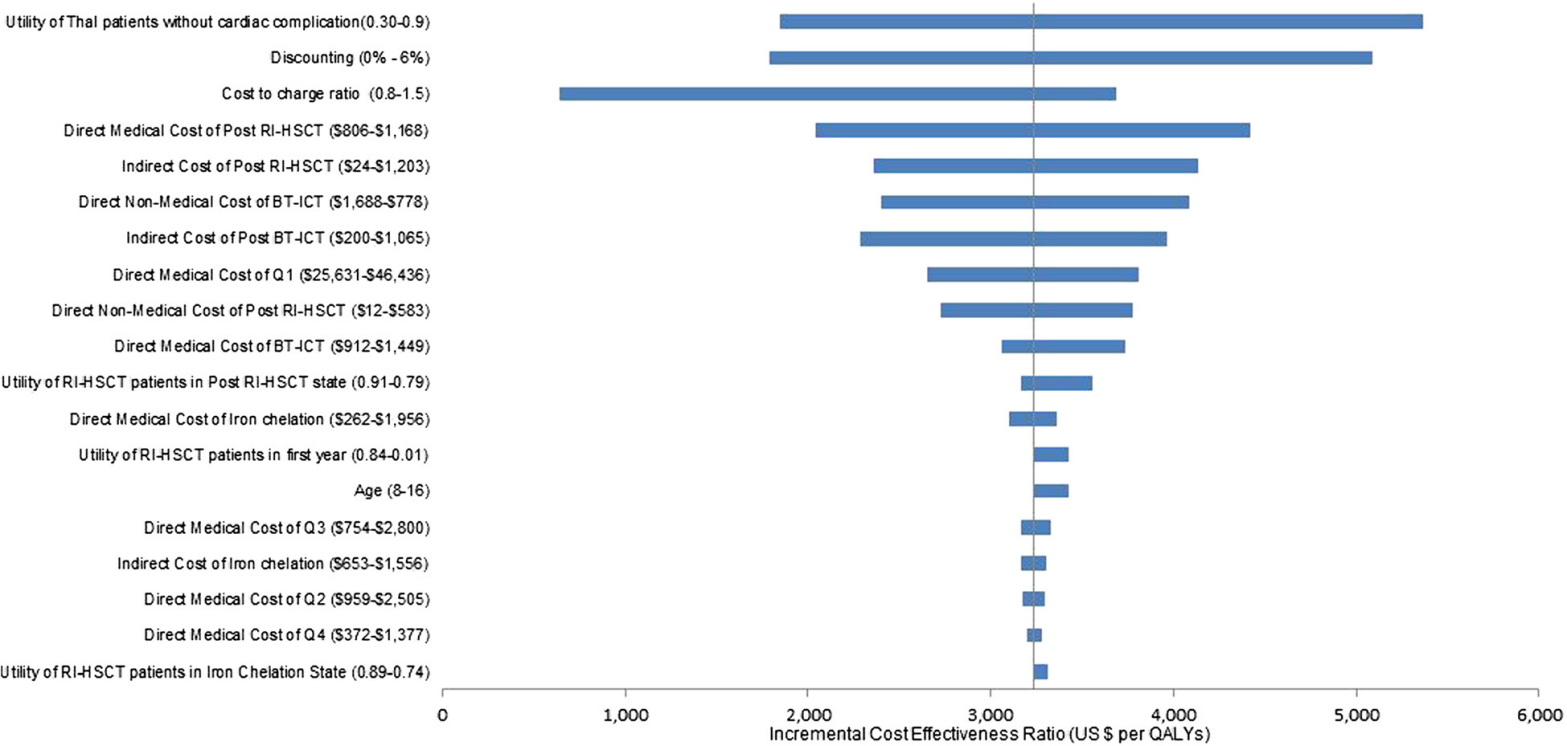
**A series of one-way sensitivity analyses comparing RI-HSCT to BT-ICT.** RI-HSCT: Reduced intensity hematopoietic stem cell transplantation; BT-ICT: Blood transfusion combined with iron chelating therapy.

A series of one-way sensitivity analyses as shown in Figure 3 showed that the most influential parameter was utility of severe thalassemia patients without cardiac complication patients. When varying the utility of severe thalassemia patients without cardiac complication patients from 0.3 to 0.9, the cost-utility ratio was shifted to US$ 1,843 and 5,364. Discounting rate was the second influential parameter.

When varying the discounting rate from 3% to 0% and 6%, the cost-utility value was shifted to US$ 1,792 and 5,087, respectively. When varying the cost to charge ratio from 0.8 to 1.5, the cost-utility ratio was shifted to US$ 641 and 3,689.

**Figure 4:**
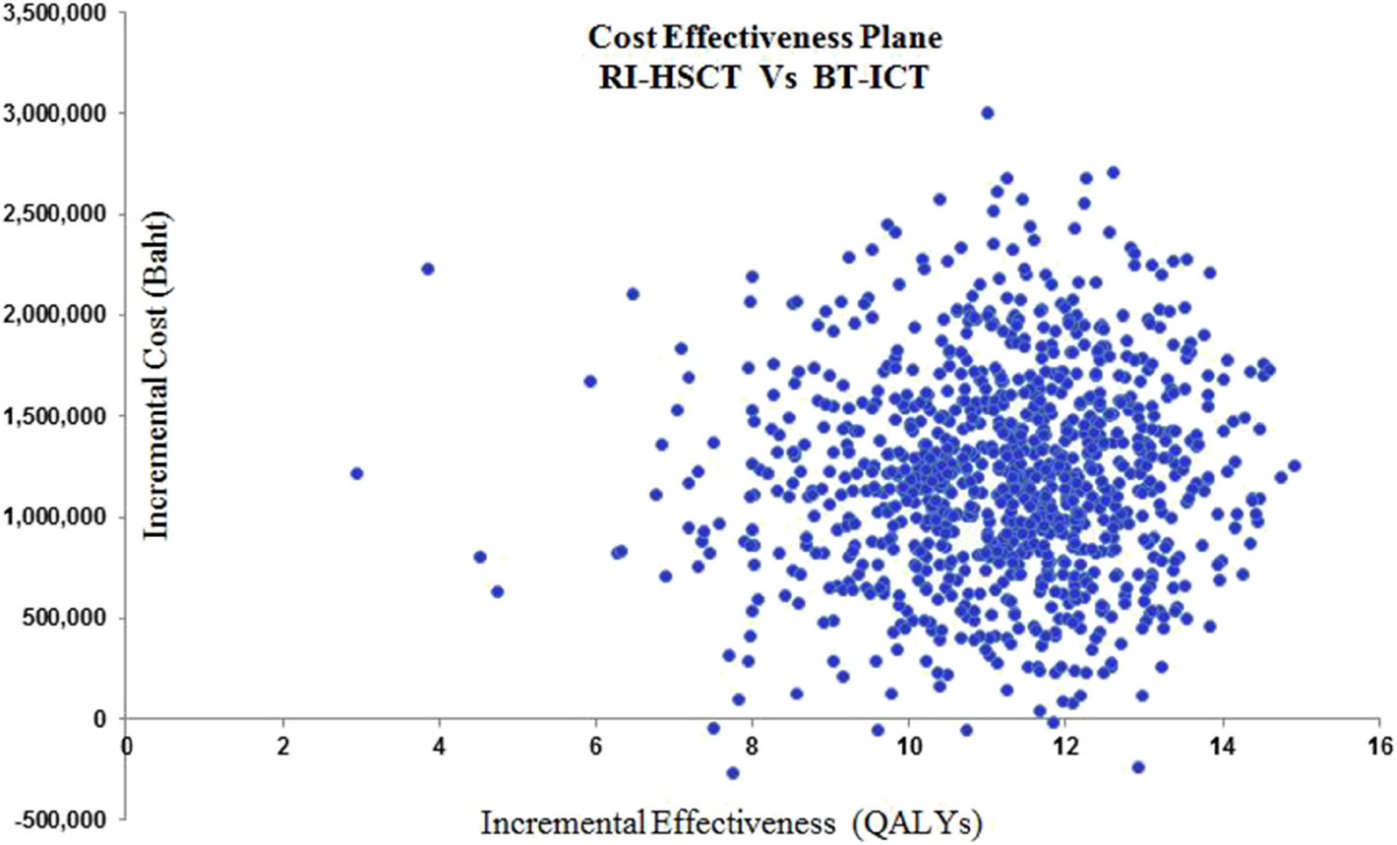
**Probabilistic sensitivity analysis of the incremental costs and effectiveness (QALYs) for RI-HSCT vs BT-ICT presented on a cost-effectiveness plane.** RI-HSCT: Reduced intensity hematopoietic stem cell transplantation; BT-ICT: Blood transfusion combined with iron chelating therapy; QALYs: quality adjusted life-years.

**Figure 5:**
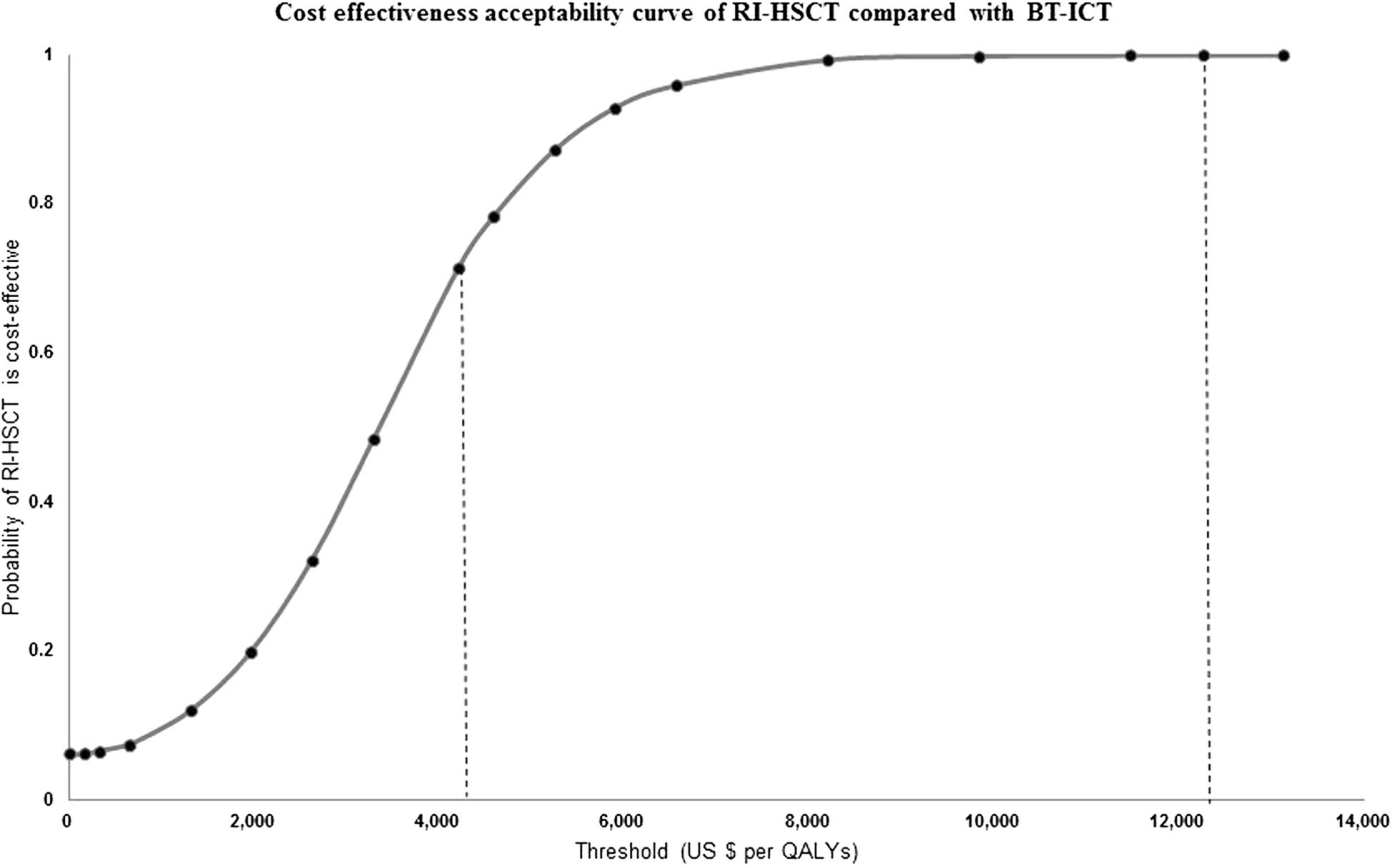
**Cost effectiveness acceptability curve showing the probability that using RI-HSCT is cost-effective compared with BT-ICT.** RI-HSCT: Reduced intensity hematopoietic stem cell transplantation; BT-ICT: Blood transfusion combined with iron chelating therapy.

The result of 1,000 simulations of PSA showed that RI-HSCT was estimated to have higher cost and more effective compared to conventional therapy (BT-ICT) (Figure 4). Cost effectiveness acceptability curves (CEAC) showed the threshold values of US$ 4,210 (128,403 THB) and US$ 12,263 (385,209 THB), RI-HSCT have 71.3% and 99.9%, respectively of being cost-effective when compared with BT-ICT (Figure 5).

## Discussion

Our findings demonstrate that RI-HSCT is highly effective and very cost-effective for adolescent and adult thalassemia patients given the consideration of local context on the willingness to pay value. The high success rate of this transplantation is highly regarded as a strong evidence for considering RI-HSCT as a therapeutic alternative for this population. On the overall basis, this study provides key relevant information aiding policy makers to make informed decision making regarding resource allocation.

Clinical effectiveness of RI-HSCT is a key factor driving this intervention to be cost-effective. The high effectiveness rate, demonstrated as low treatment failure and high survival rate, is attributable to 2 key steps of RI-HSCT. First, we used rigorous criteria to select patients for RI-HSCT. We selected only patients with consistent blood transfusion and iron chelation therapy for 1–2 years before transplantation. Second, we had a good pre-transplantation management which was the use of effective medications to suppress bone marrow expansion or alloimmunization from previous multiple blood transfusions such as hydroxyurea, fludarabine and dexamethasone. Previous studies showed that these medications could increase the success rate of transplantation [39]. At present, the moving target of thalassemia treatment is focused on transplantation; a number of cost-effectiveness studies of this treatment have further improved. While the improvement in non-transplant therapy has been a relatively slower process.

The findings of being cost-effective are robust across all sensitivity analyses performed. Our study revealed that the ICERs were sensitive to utility values of BT-ICT patients, discount rate and cost to charge ratio. However, there remains unchanged in terms of direction of overall research findings. The cost-effectiveness acceptability curves suggest that the probability that the RI-HSCT being cost-effectiveness compared to usual care is approximately sixty-four percent using one GDP and ninety-nine percent when using three GDP per capita.

To our knowledge, our study is the first study that determine the cost-effectiveness of RI-HSCT in adolescence and young adult with severe thalassemia patients. A number of clinical studies of RI-HSCT have demonstrated its effectiveness in both pediatric and adult thalassemia population [6-8], but there is a lack of economic evidence of RI-HSCT. There was only one previously published cost-effectiveness study of HSCT [29]. They reported that HSCT to severe thalassemia patients with related or un-related donors was likely to be cost-effective only when provided to patients aged up to 10 years. Our study had demonstrated that RI-HSCT may be a viable option for adolescent and adult severe thalassemia patients who are older than 10 due to the high clinical benefits and cost-effective treatment.

We believe that our findings are highly valid and contextually relevant because we used local data as much as possible in our analysis, as illustrated by the following examples. First, even though there is a lack of survival data for patients treated with blood transfusions in Thailand, we adjusted the mortality rate of BT-ICT patients [33] by incorporating Thai age-specific mortality rate to reflect Thai population. Secondly, we obtained data on direct medical costs, direct non-medical costs and indirect costs of patients receiving BT-ICT from one of the largest cohort studies of thalassemia patients in Thailand [28,29]. Thirdly, all cost data were acquired from reliable local sources i.e. national reimbursement rate specified by MOPH and Drugs and Medical Supplies Information Center (DMSIC), Ministry of Public Health. Most importantly, our study was conducted in accordance with pharmacoeconomic guideline in Thailand [9]. The societal perspective undertaken in our analysis was the most widely recommended perspective. Fourth, to our knowledge, this study is the first study in Thailand directly collected quality of life from RI-HSCT patients. Moreover, we derived utility values using Thai preference score for EQ-5D. These make our results more reliable for Thai context.

A number of limitations in our study deserved discussion. First, the survival data and transition probabilities for RI-HSCT patients as well as the direct medical costs of RI-HSCT were obtained from the small number of patients. Because RI-HSCT is considered a new and innovative treatment of thalassemia in Thailand, it was offered only to a few numbers of patients receiving care at a couple of hospitals in Bangkok. Second, the sensitivity analysis indicated that the ICER per QALY gained was most sensitive to changes in the utility of BT-ICT patients which this study obtained from foreign data (UK). This is identified as an area where further studies using local data are needed. Third, the direct medical costs of the interventions were obtained from a single hospital. RI-HSCT is the innovation intervention to cure thalassemia patients and need expert to look after these patients. RI-HSCT patients need to be followed up regularly at the same hospital so all costs could be collected in a single hospital database. However, using a single hospital database could however underestimate the true costs of blood transfusions, as patients might receive these in a number of different hospitals.

These findings are very favorable and could be interpreted by policy makers as paramount evidence to strongly endorse the decision to support the program; however, most of the decision making generally cannot be made based solely on a cost-effectiveness analysis.

Before deciding to support to reimburse RI-HSCT in the any health benefit package, decision makers may be interested in knowing other important issues about the characteristics of the populations that benefit from the transplantation. Then, budget impact analysis should be done along with the implementation suggested, in order to estimate the total budget needed. Moreover, preparing health care system including facilities, resources and treatment knowledge should be transferred to various centers to further expand the chance for the patients to access the treatment. In addition, it is crucial to review the reimbursement system for transplantation in other countries in order to develop appropriate package for the Thai context.

## Conclusion

RI-HSCT is considered a curative option for adolescence and young adult with severe thalassemia as it is highly effective and cost-effective throughout the patient’s lifetime. Clinicians and policy makers may consider including RI-HSCT in the health-benefit package given the long-term value for money of this intervention.

## Abbreviations

RI-HSCT: Reduced intensity hematopoietic stem cell transplantation;BT-ICT: Blood transfusions combined with iron chelating therapy;SF-36: Short-form 36;QoL: Quality of life;EQ-5D: European quality of life – 5 dimensions;QALYs: Quality adjusted life- years;ICER: Incremental cost-effectiveness ratio;PSA: Probabilistic sensitivity analyses;GDP: Gross domestic product;PBSC: Peripheral blood stem cell;CPI: Consumer price index, Pre-BMT, pre-bone marrow transplantation;WTP: Willingness to pay;CCR: Cost to charge ratio;NMB: Net monetary benefit

## Competing interests

The authors declare that they have no competing interests.

## Authors’ contributions

RS participated in design of the study, developed the cost-effectiveness model, conducted the cost-effectiveness analyses, drafted the manuscript and critically reviewed the manuscript for important intellectual content. NC participated in design of the study, developed the cost-effectiveness models, conducted the cost-effectiveness analysis, and critically reviewed the manuscript for important intellectual content. SP participated in the design of the study, the acquisition of data for use in the cost-effectiveness models and critically reviewed the manuscript for important intellectual content. SS participated in the acquisition of data for use in the cost-effectiveness models and critically reviewed the manuscript for important intellectual content. NS participated in the acquisition of data for use in the cost-effectiveness models and critically reviewed the manuscript for important intellectual content. UB participant in the design of the study and the acquisition of data for use in the cost-effectiveness models and critically reviewed the manuscript for important intellectual content. SH participated in design of the study and critically reviewed the manuscript for important intellectual content. All authors have read and approved the final manuscript.

## Pre-publication history

The pre-publication history for this paper can be accessed here:

http://www.biomedcentral.com/1472-6963/13/45/prepub
